# Preparation and Characterization of Stealth Archaeosomes Based on a Synthetic PEGylated Archaeal Tetraether Lipid

**DOI:** 10.1155/2011/396068

**Published:** 2011-03-21

**Authors:** Julie Barbeau, Sandrine Cammas-Marion, Pierrick Auvray, Thierry Benvegnu

**Affiliations:** ^1^Ecole Nationale Supérieure de Chimie de Rennes, UMR 6226 CNRS, Avenue du Général Leclerc, CS 50837, 35708 Rennes Cedex 7, France; ^2^Université européenne de Bretagne, France; ^3^C-RIS Pharma, Parc Technopolitain, Atalante Saint-Malo, 35400 Saint-Malo, France

## Abstract

The present studies were focused on the formation and characterization of sterically stabilized archaeosomes made from a synthetic PEGylated archaeal lipid. In a first step, a synthetic archaeal tetraether bipolar lipid was functionalized with a poly(ethylene glycol), PEG, and (PEG_45_-Tetraether) with the aim of coating the archaeosome surface with a sterically stabilizing hydrophilic polymer. In a second step, Egg-PC/PEG_45_-Tetraether (90/10 wt%) archaeosomes were prepared, and their physicochemical characteristics were determined by dynamic light scattering (size, polydispersity), cryo-TEM (morphology), and by high-performance thin layer chromatography (lipid composition), in comparison with standard Egg-PC/PEG_45_-DSPE formulations. Further, a fluorescent dye, the carboxyfluorescein, was encapsulated into the prepared archaeosomes in order to evaluate the potential of such nanostructures as drug carriers. Release studies have shown that the stability of Egg-PC/PEG_45_-Tetraether-based archaeosomes is significantly higher at 37°C than the one of Egg-PC/PEG_45_-DSPE-based liposomes, as evidenced by the slower release of the dye encapsulated into PEGylated archaeosomes. This enhanced stability could be related to the membrane spanning properties of the archaeal bipolar lipid as already described with natural or synthetic tetraether lipids.

## 1. Introduction

In the drug-delivery field, several nanocarriers have been proposed to improve the therapeutic index of various biologically active molecules such as peptides. Indeed, *in vivo* administration of peptides is still limited by their poor bioavailability and susceptibility to cleavage by proteases. In order to obtain a satisfactory therapeutic effect, the peptide has to be frequently administrated at high doses leading to unwanted toxic effects, such as induction of immune response. Consequently, peptide encapsulation into site-specific delivery systems can offer solutions to the above-mentioned problems. Indeed, the nanocarriers can (i) enhance drug solubility, (ii) control drug release thus avoiding toxic side effects, (iii) improve drug biodistribution, (iv) and, if appropriate molecule is grafted on the nanocarrier surface, target a specific site of action. Several nanovectors have been used to encapsulate various therapeutic peptides such as liposomes, nanoparticles, and nano- or microgels [1–8]. Among these nanocarriers, liposomes are of great importance because of their relatively large carrying capacity and the possibility to entrap either hydrophilic, hydrophobic, or amphiphilic drugs. Moreover, a good knowledge of such vectors has been acquired since the first discovery of liposomes by Bangham and Horne [9] attested by commercially available anticancer liposomial formulations such as Doxil [10, 11]. However, despite encouraging results, a major limitation to the development of liposomes as drug carriers is their instability, especially during their transit to the site of action [12]. Attempts to improve their stability, either by incorporation of high amount of cholesterol or by coating the liposome surface with poly(ethylene glycol), have led to limited success. 

Within this context, archaeosomes, made with one or more of either the ether lipids found in Archaea bacteria or synthetic archaeal lipids, constitute a novel family of liposomes exhibiting higher stabilities in several conditions, such as high temperature, alkaline or acidic pH, presence of phospholipases, bile salts, and serum media [13, 14]. Therefore, because of their biocompatibility and higher stability, archaeosomes have been extensively studied for potential applications as drug/gene and vaccine delivery systems [14, 15].

Over the last decade, our research group has developed synthetic analogues of natural archaeal tetraether lipids and studied their uses in cationic archaeosome formulations as efficient gene delivery systems [16–18]. Our next objective was to evaluate the potential applications of archaeosome technology for the delivery of additional hydrophilic substrates such as antitumoral peptides (Project Sealacian: encapsulation of natural marine peptides, extracted from *Scyliorhinus canicula*, for their site-specific delivery). Our attention was then directed towards the preparation and the formulation of a PEGylated archaeal tetraether lipid (PEG_45_-Tetraether) to provide neutral coated archaeosomes valuable as peptide nanocarriers. In order to assess the value of this new family of stealth liposomes, physicochemical characteristics (DLS, cryo-TEM, and HPTLC), dye encapsulation and release profile for a PEGylated archaeosome formulation were determined and compared to those measured from a conventional PEGylated liposome formulation.

## 2. Materials and Methods

### 2.1. Materials

Egg-PC was purchased from Sigma. 1,2-distearoyl-*sn*-glycero-3-phosphatidylethanolamine-*N*-[methoxy-poly(ethylene glycol)-2000], ammonium salt, (PEG45-DSPE) was purchased from Aventi Polar. PEG45-Tetraether was synthesized according to a four-step procedure from the tetraether diol **1** available in our laboratory [13]. All reactions were carried out under nitrogen atmosphere with dry, freshly distilled solvents under anhydrous conditions. Dichloromethane (CH_2_Cl_2_) and methanol (MeOH) were distilled over calcium hydride. All other reagents were used directly from the supplier without further purification unless noted. Analytical thin-layer chromatography (TLC) was performed on Merck 60 F_254_ silica gel nonactivated plates. A solution of 5% H_2_SO_4_ in EtOH or ultraviolet fluorescence was used to develop the plates. Column chromatography was performed on silica gel MERCK 60 H (5–40 *μ*m). Nuclear magnetic resonance spectra (^1^H NMR and ^13^C NMR) were recorded on a Brucker ARX 400 instrument (^1^H at 400 MHz, ^13^C at 100 MHz). Data are reported as follows: chemical shift (number of hydrogen, multiplicity, and coupling constants if applicable). The chemical shifts (*δ*) are reported as parts per million (ppm) referenced to the appropriate residual solvent peak. Coupling constants are reported in Hertz (Hz). Abbreviations are as follows: *s* (singlet), *d* (doublet), *t* (triplet), *q* (quartet), *dd* (doublet of doublet), and *m* (multiplet). High-resolution mass spectra (HRMS) were performed by CRMPO (Université de Rennes 1) on a MS/MS ZabSpec TOF Micromass. Accurate masses are reported for the molecular ions [M+H]^+^, [M+Na]^+^, [M+K]^+^, or [M−H]^−^. Optical rotations were measured on a Perkin-Elmer 341 polarimeter. IR spectra were recorded on a Nicolet 250 FT-IR spectrometer.

HPTLC plates (20∗10 cm, silica gel 60, 0.2 mm layer thickness, Nano-Adamant UV_254_) were purchased from Macherey-Nagel. Before use, the HPTLC plates were prewashed with methanol, dried on a CAMAG TLC plate heater III at 120°C for 20 min, and kept in an aluminum foil in a desiccator at room temperature. All solvents were of HPTLC grade.

### 2.2. Synthesis of PEG_45_-Tetraether

**1-*O-*acetyl-2,2′-di-*O-*(3,7,11,15-tetramethylhexadecyl)-3,3′-*O*-(1,32-(13,20-dioxa)-dotriacontane-(*cis-*15,18-methyliden))diyl-di-*sn*-glycerol 2**

A mixture of tetraether diol **1** (600 mg, 0.495 mmol, 1 equiv.), acetic anhydride (151 *μ*L, 3.5 equiv.) and sodium acetate (41 mg, 1 equiv.) was stirred under reflux for 24 h. Water was added and the aqueous phase was extracted twice with CH_2_Cl_2_. The combined organic phases were dried (MgSO_4_) and concentrated under reduced pressure. The residue was purified by flash chromatography on silica gel (petroleum ether (PE)/AcOEt: 98 : 2) to yield the monoacetate derivative **2** (305 mg, 49%) as a colorless oil. *R*_*f*_ = 0.15 (PE/AcOEt: 9 : 1). [*α*]^20^_D_ : +9° (*c* 1.0, CHCl_3_). FT-IR *υ* (cm^−1^) 2924 (CH_3_), 2853 (CH_2_), 1746 (CO), 1463 (CH_2_), 1377 (CH_3_), 1115 (COC); ^1^H NMR (CDCl_3_, 400 MHz) *δ* 0.80–0.89 (31H, m), 1.02–1.81 (92H, m), 1.91–1.98 (1H, m), 2.07 (3H, s), 2.13–2.23 (2H, m), 3.29 (4H, d, *J* = 6.9 Hz), 3.39 (4H, *t*, *J* = 6.7 Hz), 3.43 (4H, *t*, *J* = 6.6 Hz), 3.44–3.74 (m, 8H), 4.11 (1H, dd, *J* = 5.7, 11.6 Hz), 4.22 (1H, dd, *J* = 4.1, 11.6). ^13^C NMR (CDCl_3_, 100 MHz) *δ* 19.61, 19.68, 19.75, 20.93, 22.63, 22.72, 24.32, 24.46, 24.48, 24.81, 26.13, 28.02, 29.53, 29.62, 29.71, 29.79, 30.03, 31.61, 32.81, 33.01, 36.73, 37.22, 37.33, 37.38, 37.43, 37.51, 38.79, 39.38, 40.12, 40.68, 63.12, 64.13, 68.61, 68.89, 68.91, 70.16, 70.19, 70.6, 70.9, 71.7, 71.9, 75.6, 76.5, 78.6, 170.9. HRMS (ESI) calcd. for C_79_H_157_O_9_ (M+H)^+^ 1250.1827, found 1250.1823; HRMS (ESI) calcd. for C_79_H_156_O_9_Na [M+Na]^+^ 1272.1647, found 1272.1650; HRMS (ESI) calcd. for C_79_H_156_O_9_K [M+K]^+^ 1288.1386, found 1288.1381.

**1-*O-*acetyl-1′-carboxy-2,2′-di-*O-*(3,7,11,15-tetramethylhexadecyl)-3,3′-*O*-(1,32-(13,20-dioxa)-dotriacontane-(*cis-*15,18-methyliden))-diyl-di-*sn*-glycerol 3**

To a solution of alcohol **2** (50 mg, 0.04 mmol, 1 equiv.) in AcOEt (1 mL), a 0.5 M aqueous solution of KBr (8 *μ*L, 0.1 equiv.) and TEMPO (1 mg, 0.2 equiv.) were added. At 0°C, a 5% aqueous solution of NaOCl (69 *μ*L) was then added dropwise. The reaction mixture was stirred at room temperature for 2 h, the solution was acidified until pH 3-4 using 5% HCl and a 25% aqueous solution of NaO_2_Cl (17 *μ*L) was added slowly. After stirring for 3 h at room temperature, the mixture was extracted with AcOEt, washed with a saturated aqueous solution of NaCl, dried (MgSO_4_), and concentrated under reduced pressure to give the carboxylic acid derivative **3** (45 mg, 90%) as a colorless oil. *R*_*f*_ = 0.28 (CH_2_Cl_2_/CH_3_OH: 9 : 1). FT-IR *υ* (cm^−1^) 2924 (CH_3_), 2853 (CH_2_), 1746 (COCH_3_), 1733 (COOH), 1463 (CH_2_), 1377 (CH_3_), 1115 (COC); ^1^H NMR (CDCl_3_, 400 MHz) *δ* 0.80–0.89 (31H, m), 1.02–1.81 (92H, m), 1.91–1.98 (1H, m), 2.07 (3H, s), 2.13–2.23 (2H, m), 3.29 (4H, d, *J* = 6.9 Hz), 3.39 (4H, *t*, *J* = 6.7 Hz), 3.41–3.72 (m, 12H), 3.79 (1H, ddd, *J* = 1.0, 3.3, 10.5 Hz), 4.03 (1H, dd, *J* = 4.1, 11.6 Hz), 4.11 (1H, d, *J* = 5.7, 11.6 Hz), 4.22 (1H, dd, *J* = 4.1, 11.6 Hz). ^13^C NMR (CDCl_3_, 100 MHz) *δ* 19.61, 19.68, 19.75, 20.91, 22.63, 22.72, 24.3, 24.46, 24.48, 24.81, 26.11, 28.02, 28.79, 29.51, 29.62, 29.73, 29.82, 30.02, 31.59, 32.82, 33.01, 36.68, 36.81, 36.93, 37.04, 37.12, 37.19, 37.33, 37.38, 37.41, 37.52, 38.84, 39.37, 40.12, 40.66 63.08, 63.12, 64.15, 68.65, 68.89, 68.91, 70.16, 70.19, 70.63, 70.91, 71.72, 71.91, 75.57, 76.53, 78.59, 170.91, 171.88. HRMS (ESI) calcd. for C_79_H_153_O_10_ [M−H]^−^ 1262.1463, found 1262.1447.

**PEG_45_-Tetraether**

To a solution of carboxylic acid **3** (16.6 mg, 0.015 mmol, 1 equiv.) and TBTU (4.6 mg, 1 equiv.) in dry CH_2_Cl_2_ (1 mL) was added DIEA (3.4 *μ*L, 1.3 equiv.) under a nitrogen atmosphere. After 20 min at room temperature, a solution of H_2_N-PEG_45_-OMe** 4 **(24.4 mg, 1 equiv.) in dry CH_2_Cl_2_ (2 mL) was added and the reaction mixture was stirred under reflux for 12 h. A few drops of a 5% HCl aqueous solution were then added and the solvents were removed under reduced pressure. The residue was dissolved in CHCl_3_ (1 mL) and purified on a Sephadex LH-20 column eluting with a mixture of CHCl_3_/CH_3_OH (9 : 1) to give a white solid (41 mg, 80%) composed of the expected monoacetate derivative **5** and the starting H_2_N-PEG_45_-OMe **4 **in a 80 : 20 ratio. FT-IR *υ* (cm^−1^) 2924 (CH_3_), 2855 (CH_2_), 1746 (COCH_3_), 1651 (CONH), 1103 (COC); ^1^H NMR (CDCl_3_, 400 MHz) *δ* 0.82–0.86 (31H, m, 10 CH_3_), 1.00–1.80 (92H, m), 1.91–1.98 (1H, m), 2.06 (3H, s), 2.13–2.23 (2H, m), 3.27 (4H, d, *J* = 6.9 Hz), 3.36–3.58 (23H, m), 3.37 (3H, s), 3.59–3.68 (169H, m), 3.73–3.77 (1H, m), 3.81 (1H, dd, *J* = 4.1, 5.6 Hz), 3.88 (1H, dd, *J* = 2.5, 6 Hz), 4.08 (1H, dd, *J* = 5.6, 11.6 Hz), 4.21 (1H, dd, *J* = 4.1, 11.6 Hz), 7.03 (1H, m). ^13^C NMR (CDCl_3_, 100 MHz) *δ* 14.08, 19.58, 19.65, 19.72, 20.91, 22.60, 22.69, 24.32, 24.45, 24.77, 26.03, 26.07, 26.15, 27.93, 28.82, 29.32–29.84, 32.76, 33.9, 36.84–37.50, 38.57, 39.33, 39.70 59.00, 62.97, 68.60, 69.74, 69.83, 70.29, 70,53, 70.91, 71.53, 71.56, 71.69, 71.83, 71.89, 75.60, 77.20, 78.21, 80.50, 170.53, 170.72. To a solution of this white solid (41 mg) in a CH_2_Cl_2_/CH_3_OH (1 : 1) mixture, was added a freshly prepared solution of CH_3_ONa in CH_3_OH (0.1 M, 1 equiv.). The reaction mixture was stirred at room temperature for 4 h. Amberlite resin (IR120) was added, the reaction mixture was filtered, and the solvents were evaporated under reduced pressure. A white powder was isolated (41 mg) composed of the desired PEG_45_-Tetraether and the starting H_2_N-PEG_45_-OMe** 4** in a 80 : 20 ratio. *R*_*f*_ = 0.28 (CHCl_3_/CH_3_OH/H_2_O: 9 : 1). FT-IR *υ* (cm^−1^) 2927 (CH_3_), 2855 (CH_2_), 1652 (CONH), 1103 (COC); ^1^H NMR (CDCl_3_, 400 MHz) *δ* 0.82–0.86 (31H, m, 10 CH_3_), 1.00–1.80 (92H, m), 1.91–1.98 (1H, m), 2.13–2.23 (2H, m), 3.27 (4H, d, *J* = 6.9 Hz), 3.36–3.58 (23H, m), 3.37 (3H, s), 3.59–3.68 (169H, m), 3.73–3.77 (1H, m), 3.81 (1H, dd, *J* = 4.1, 5.6 Hz), 3.88 (1H, dd, *J* = 2.5, 6 Hz), 7.03 (1H, m). ^13^C NMR (CDCl_3_, 100 MHz) *δ* 14.09, 19.58, 19.65, 19.72, 22.60, 22.69, 24.32, 24.45, 24.77, 26.03, 26.07, 26.15, 27.93, 28.82, 29.32–29.84, 32.76, 33.9, 36.84–37.50, 38.57, 39.33, 39.70 59.00, 62.97, 68.60, 69.74, 69.83, 70.29, 70,53, 70.91, 71.53, 71.56, 71.69, 71.83, 71.89, 75.60, 77.20, 78.21, 80.50, 170.52.

### 2.3. Preparation of PEGylated Archaeosomes and PEGylated Liposomes

Stock solutions of Egg-PC (1 mg/mL) and PEG_45_-DSPE (1 mg/mL) were prepared in CHCl_3_ : CH_3_OH (2 : 1, v/v), while stock solutions of PEG_45_-Tetraether (1 mg/mL) were prepared in CHCl_3_. 

Liposomes and archaeosomes were obtained by the hydration method as already described elsewhere [16–18]. Briefly, the selected lipid solutions were mixed to yield either a mixture of Egg-PC and PEG_45_-DSPE (90 : 10 wt%) or a mixture of Egg-PC and PEG_45_-Tetraether (90 : 10 wt%) with a total lipid concentration of 1 mg/mL. The organic solvents were then evaporated using a rotary evaporator, and the lipid films thus obtained were dried under high vacuum for 2 hours at room temperature. The dried lipid films were then hydrated with 1 mL of milliQ water. The solutions were vortexed and left at 4°C overnight. Archaeosome or liposome formulations were sonicated at room temperature for two times 5 min with interval of 5 min using a Fischer scientific sonication bath (FB 15051) at 80 KHz. Each formulation was realized in duplicate.

### 2.4. Encapsulation of Carboxyfluorescein into PEGylated Archaeosomes

PEG_45_-Tetraether (90 : 10 wt%) based archaeosomes and Egg-PC/PEG_45_-DSPE (90 : 10 wt) *PEGylated Liposomes*: Carboxyfluorescein (CF) was encapsulated in Egg-PC based liposomes during the hydration phase as described elsewhere [19]. Briefly, Egg-PC/PEG_45_-Tetraether (90 : 10 wt%) and Egg-PC/PEG_45_-DSPE (90 : 1 wt%) lipid films were prepared as described above. After drying, both lipid films were hydrated with 1 mL of a tris(hydroxyl methyl) methylamine buffer (Tris buffer) at pH 7.4 containing CF at a concentration of 100 mM. The solutions were vortexed and left at 4°C overnight. Both PEGylated archaeosomes and PEGylated liposomes containing CF were sonicated (Fischer scientific sonication bath FB 15051-80 KHz) at room temperature for two times 5 min with interval of 5 min. Nonencapsulated CF was eliminated by size exclusion column chromatography on the Sephadex G-50 gel with the Tris buffer as eluent. Both PEGylated archaeosomes and PEGylated liposomes containing CF were analyzed by DLS and by fluorescence using a Fluoromax-3 (Horiba) spectrofluorimeter with excitation and emission wavelengths of 490 and 515 nm, respectively.

### 2.5. Size, Polydispersity, and Zeta Potential Measurements

The size (average diameter obtained by the cumulant result method), polydispersity and zeta potential of the formulations were measured by dynamic light scattering using a Delsa Nano Beckman Coulter apparatus at 25°C. The samples were diluted 2 times with milliQ water.

### 2.6. Cryo-TEM Measurements

The cryo-TEM analysis of PEGylated liposomes and PEGylated archaeosomes was realized by Dr. Olivier LAMBERT at the University of Bordeaux (Group “Chimie et Biologie des Membranes et Nano-objets”, UMR 5248 CNRS).

Each sample (5 *μ*L) was deposited on a grid covered with a carbon film having 2 *μ*m diameter holes previously exposed to treatment with UV-ozone. The excess of water was removed by absorption with filter paper to form a thin layer of water suspended inside the holes. This grid was then plunged quickly (EM CPC, Leica) in liquid ethane (−178°C). Rapid freezing of the thin layer of liquid water in vitreous ice (absence of crystals) preserved biological structures. Grids were then placed in a suitable object carrier for observing the samples at −170°C. Observation under a microscope (FEI Tecna F20) was carried out in the mode low dose, limiting the effects of beam irradiation on the lipid material. Images were recorded using an ultrasensitive camera (Gatan, USC 1000) 2K∗2K with pixel size of 14 *μ*m. The electron dose used was 10–20 electrons/Å^2^. The image resolution under these conditions was about 2 nm.

### 2.7. Lipid Composition of Liposomes and Archaeosomes by HPTLC

The lipid compositions of formulations were determined after ultrafiltration. The samples were filtered through 10 000 NMWL pore filters (Micron YM-10, Millipore Corporation) by ultracentrifugation at 15 000 g for 1 hour at 15°C. The supernatants were recovered, lyophilized, dissolved in 1 mL of methanol, and analyzed by HPTLC using the automated HPTLC system from CAMAG (Muttenz, Switzerland). The samples, the appropriate lipid standard solutions and a blank solution composed by pure methanol were spotted on 20 × 10 cm HPTLC plates using the Automatic TLC Sampler 4 from CAMAG (Muttenz, Switzerland). Each lane was spotted 10 mm above the bottom edge of the plate and was 6 mm length with 17 mm spacing between lanes. The spotting volume was 10 *μ*L or 20 *μ*L. A maximum of 20 lanes was spotted on a single plate. After evaporation of the sample solvent, the plates were developed in a closed twin trough chamber for 20∗10 cm plates (CAMAG) containing 10 mL of the mobile phase (CHCl_3_/MeOH/H_2_O, 18/4/0.5) in each trough. The chamber was pre-equilibrated at least 20 min before the development. The development was stopped when the solvent had migrated 80 mm. The plates were dried on a CAMAG TLC plate heater III at either 60°C for 30 min. The HPTLC plates were postchromatographic derivatizated by dipping 5 s into a primuline solution (5 mg of primuline in 100 mL of acetone/H_2_O (80/20) mixture). HPTLC plates were then dried at room temperature for 10 min and at 60°C for 30 min on a CAMAG TLC plate heater III. Plates were then scanned from 6 mm above the bottom edge of the plate to the solvent front, using a CAMAG TLC scanning densitometer. The measurements were performed in fluorescence mode at *λ* = 366 nm with a scanning speed of 20 mm/s, a slit dimension of 4∗0.2 mm (Micro) and deuterium and tungsten lamps. Data were stored online on a personal computer, and integration as well as quantification was performed with the software package CATS from CAMAG. Calibration was performed by applying standard solutions in concentration given below:

Egg-PC (*R*_*f*_ = 0.04): 10 *μ*g, 7.5 *μ*g, 5 *μ*g, and 2.5 *μ*g,
PEG_45_-DSPE (*R*_*f*_ = 0.46): 2 *μ*g, 1 *μ*g, 0.5 *μ*g, and 0.25 *μ*g,
PEG_45_-Tetraether (*R*_*f*_ = 0.79): 2 *μ*g, 1 *μ*g, 0.5 *μ*g, and 0.25 *μ*g.

Peak heights and peak areas were used for quantification. Calibration curves were calculated for each lipid or archaeal lipid, with a linear regression mode. In order to reduce experimental errors, individual calibration curves were obtained for every HPTLC plate. The amount of Egg-PC and PEG_45_-DSPE in liposomes, after ultrafiltration, and of Egg-PC and PEG_45_-Tetraether in archaeosomes, after ultrafiltration, were calculated from the calibration curves.

### 2.8. Carboxyfluorescein Release Profile

CF release profile from both PEGylated archaeosomes and PEGylated liposomes was measured by fluorescence using a Fluoromax-3 (Horiba) spectrofluorimeter with excitation and emission wavelengths of 490 and 515 nm, respectively. Release was studied at 4°C and 37°C. The fluorescence of both formulations was measured at T0, before (I0) and after (Imax) Triton-X-100 (2 v%) addition (total disruption of liposomial membranes) and at various times (It) until almost complete CF release at 4°C and at 37°C. Release of the incorporated dye was calculated using the following equation:

(1) $$\text{Release}\;\left(\text{\%}\right)=\frac{\text{It}-\text{I}0}{\text{Imax}-\text{I}0}*100.$$

## 3. Results and Discussion

Archaeosomes made with one or more of the ether lipids found in Archaea represent an innovative family of liposomes that demonstrate higher stabilities to several conditions in comparison with conventional liposomes. The definition of archaeosomes also includes the use of synthetically derived lipids that have the unique structure characteristics of archaeobacterial ether lipids, that is, regularly branched phytanyl chains attached via ether bonds at *sn*-2,3 glycerol carbons [15]. The lipid membrane of archaeosomes may be entirely of the bilayer form if made exclusively from monopolar archaeol (diether) lipids or a monolayer if made exclusively from bipolar caldarchaeol (tetraether) lipids, or a combination of monolayers and bilayers if made from caldarchaeol lipids in addition to archaeol lipids or standard bilayer-forming phospholipids. The large variety of lipid structures reflects the need for Archaea to adjust their core lipid structures in order to be able to ensure membrane functions despite harsh destabilizing environmental conditions (high or low temperatures, high salinity, acidic media, anaerobic atmosphere, and high pressure) [20]. 

These atypical characteristics should be particularly useful for the preparation of highly stable archaeosomes. In particular, specific archaeal lipid membrane properties have to be considered in view to optimize the performance of archaeosomes: (1) the ether linkages are more stable than esters over a wide range of pH, and the branching methyl groups help both to reduce crystallization (membrane lipids in the liquid crystalline state at ambient temperature) and membrane permeability (steric hindrance of the methyl side groups); (2) the saturated alkyl chains would impart stability towards oxidative degradation; (3) the unusual stereochemistry of the glycerol backbone (opposite to mesophilic organisms) would ensure resistance to attack by phospholipases released by other organisms; (4) the bipolar lipids span the membranes and enhance their stability properties and (5) the addition of cyclic structures (in particular five-membered rings) in the transmembrane portion of the lipids appears to be a thermoadaptive response, resulting in enhanced membrane packing and reduced membrane fluidity. 

Consequently, formulations including archaeal lipids demonstrate relatively higher stabilities to oxidative stress, high temperature, alkaline or acidic pH, action of phospholipases, bile salts, and serum media. Archaeosomes can be formed using standard procedures (hydrated film submitted to sonication, extrusion or detergent dialysis) at any temperature in the physiological range or lower, thus making it possible to encapsulate thermally labile compounds. Moreover, they can be prepared and stored in the presence of air/oxygen without any degradation. The *in vitro* and *in vivo* studies indicate that archaeosomes are safe and do not elicit toxicity in mice. Thus, the biocompatibility and the superior stability properties of archaeosomes in numerous conditions offer advantages over conventional liposomes in the manufacture and the use in biotechnology including vaccine and drug/gene delivery. 

However, to study in depth archaeolipid structure-archaeosome property relationships with a view of optimizing the performance of these unusual liposomes as gene/drug nanocarriers, sufficient amounts of pure natural lipids are required. Well-defined lipids are difficult to isolate from natural extracts, and chemical synthesis appears, therefore, as an attractive means of producing model lipids that mimic the natural lipids. Within this context, our group focused on the synthesis and the evaluation of chemically pure archaeal diether and tetraether lipids that retain some of the essential structural features of archaeal membrane lipids. These studies clearly showed the interest in developing archaeosome technology from synthetic tetraether lipids possessing neutral, zwitterionic, or cationic polar heads groups for *in vitro* and *in vivo* delivery applications of nucleic acids and drugs [13, 16–18].

In order to propose a stealth version of synthetic archaeosomes that could increase blood circulation longevity by reducing or preventing protein binding and/or by inhibiting cell binding/uptake, an additional archaeosome formulation based on a novel synthetic tetraether lipid was developed. These stealth archaeosomes could be suitable for the encapsulation and the *in vivo* delivery of various bioactive molecules including peptides which are known to be highly sensitive to enzymatic or chemical degradations. Comparative studies in terms of drug-encapsulation efficacy and formulation stability between standard PEGylated liposomes and PEGylated archaeosomes were then investigated by following the leakage of the encapsulated aqueous dye 5(6)-carboxyfluorescein as a marker. 

**Figure 1:**
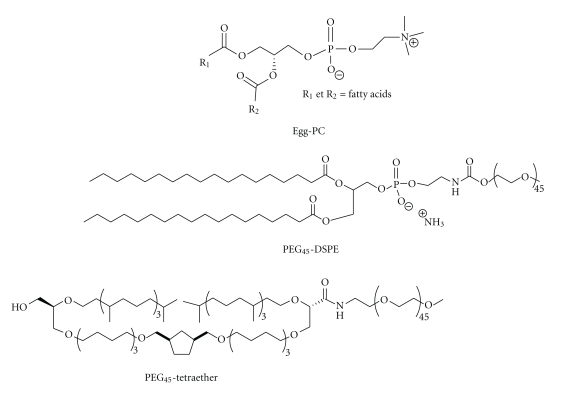
Structure of Egg-PC, PEG_45_-DSPE, and PEG_45_-Tetraether.

For that purpose, an archaeosome formulation composed by 90 wt% of a classical lipid, Egg-PC, and 10 wt% of a PEGylated tetraether archaeal lipid, PEG_45_-Tetraether (Figure 1) was selected. Indeed, previous studies relative to the use of archaeosomes as gene nanocarriers showed that the incorporation of 5 wt% to 10 wt% of tetraether archaeal lipids into bilayered vesicles led to the best efficient *in vitro* gene transfection properties [16]. In parallel, a classical liposomal formulation composed by 90 wt% of Egg-PC and 10 wt% of PEG_45_-DSPE, was prepared in order to evaluate the influence of the tetraether structure on the formulation properties in terms of stability, drug-encapsulation efficiency, and further on the *in vivo* formulation efficacy. In the present approach, the vesicle formulations were studied from a fundamental point of view, that is, through DLS and cryo-TEM measurements (size, polydispersity, and morphology), HPTLC (lipid composition), and CF release (formulation stability) in order to assess the potentiality of PEGylated archaeosomes as *in vivo* nanocarriers.

### 3.1. Synthesis of PEG_45_-Tetraether Lipid

**Scheme 1:**
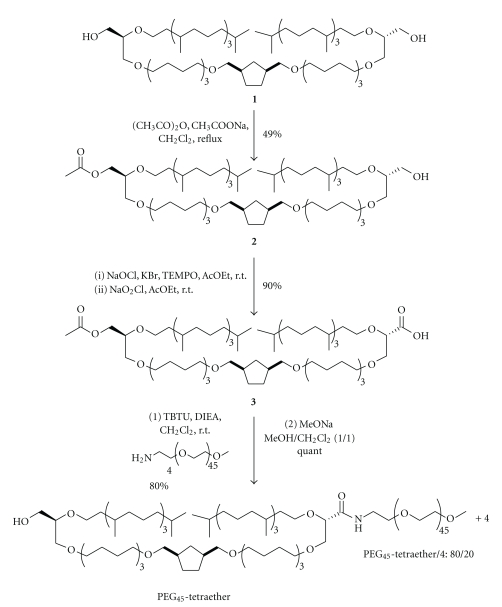
Synthesis of PEG_45_-Tetraether lipid.

The novel PEGylated archaeal lipid (PEG_45_-Tetraether) was synthesized through the functionalization of the tetraether backbone at one terminal end. The synthesis of this unsymmetrical PEGylated lipid involved the monoprotection of the starting tetraether diol **1** [13] followed by the introduction of the poly(ethylene glycol) chain (Scheme 1). The first step was carried out by an easy monoacetylation of diol **1** with sodium acetate (1 equiv.) and acetic anhydride (3.5 equiv.) to give monoacetate **2 **in a 49% yield. Alcohol **2** was then oxidized in a one-pot two-step procedure under TEMPO catalysis conditions with NaOCl and NaClO_2_ as the oxidizing agents. Fine tuning of the pH during the reaction led to a clean oxidation of **2** to carboxylic acid **3** in a yield of 90%. With acid **3** in hand, we introduced a 45-unit PEG chain using commercially available H_2_N-PEG_45_-OMe **4**. After optimization of the coupling reaction conditions, the use of the uronium salt (*O*-(benzotriazol-1-yl)1,1,3,3-tetramethyluronium tetrafluoroborate (TBTU)/ *N*,*N*′-diisopropylethylamine (DIEA) system furnished the expected PEGylated tetraether (80% yield) in addition to the starting H_2_N-PEG_45_-OMe chain (ratio: 80 : 20). It is noteworthy that the purification of the crude reaction mixture on a Sephadex LH-20 column allowed the total removal of the starting carboxylic acid **3**. The final deacylation of the hydroxyl group under Zemplèn conditions (MeONa, MeOH) gave the targeted PEG45-Tetraether lipid in a quantitative yield.

### 3.2. Physicochemical Characteristics of PEGylated Archaeosomes and PEGylated Liposomes

As described in the experimental part, formulations have been prepared using the classical lipid film hydration method followed by vesicle size reduction under sonication. The mean particle size and zeta potential of archaeosomes and liposomes were measured by dynamic light scattering. Particle mean diameters and polydispersity index are gathered in Table 1 and show that both liposomes and archaeosomes are similar in size, lower than 100 nm, with a quite narrow dispersity (around 0.30). In the same way, the mean surface potential of archaeosomes and liposomes were comparable with slightly negative values. These results are in good agreement with several reports [21, 22] that pointed out the impact of the PEG chains on liposomal size decrease and on zeta potential values close to neutrality. Most importantly, these studies revealed that the atypical structure of the tetraether did not modify the main characteristics of the resulting PEG-grafted vesicle structures (shape, size).

**Figure 2:**
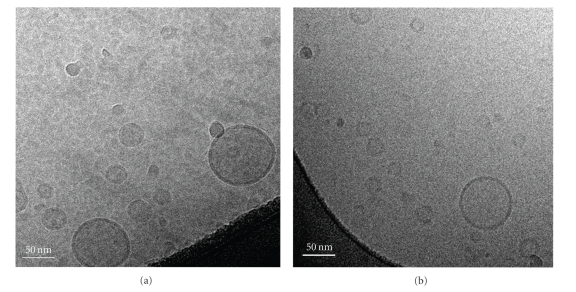
Cryo-TEM photos of (a) Egg-PC/PEG_45_-Tetraether (90 : 10 wt%) archaeosomes and (b) Egg-PC/PEG_45_-DSPE (90 : 10 wt%) liposomes. Bar is 50 nm.

Cryo-TEM was employed to investigate the morphology of the vesicles composed of PEGylated lipids. The images in Figure 2 show that PEG-bearing archaeosomes were dispersed and spherical as for classical PEGylated liposomes. The presence of an external dark circle evidenced the lipid layer surrounding the internal aqueous volume of the vesicles. It is noteworthy that no phase segregation has been evidenced meaning that the prepared formulations are quite homogenous. The sizes of the vesicles were under 100 nm and the diameter was comprised between 20 to 100 nm, which was in relatively good agreement with data obtained by DLS. Indeed, DLS measurements gave average diameters (cumulant results) lower than 100 nm with objects having diameters ranging from around 20 nm to around 200 nm.

**Figure 3:**
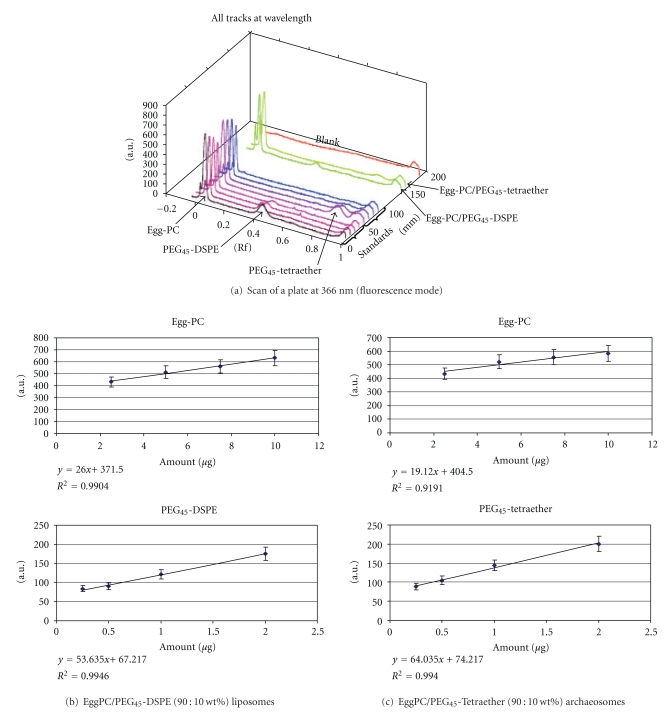
HPTLC measurements: (a) Scan of a plate at 366 nm (fluorescence mode); (b and c) standard curves, based on peak height, for each lipid composing the prepared liposomes and archaeosomes. (AU = arbitrary unit).

Besides these characteristics, it is of great interest to determine the lipid composition after formulation. For that purpose, we have used an innovative method based on quantitative thin layer chromatography, named high performance thin-layer chromatography (HPTLC). The HPTLC is a qualitative and quantitative analytical method allowing obtaining reproducible and reliable results [23]. This method is used, since several years, for analysis and quantification of lipids extracted from various sources [23–29]. More recently, the use of HPTLC has been developed for the determination of lipid compositions of liposomes [30–34] and for peptide analysis in liposomes [35]. We have, therefore, studied possibilities to use HPTLC for the determination of lipid compositions of the studied liposomes and archaeosomes. We have found conditions, described in experimental part, which allowed us to measure lipid composition. After removal of nonaggregated lipids, the supernatants were lyophilized and solubilized in methanol in order to disrupt the nanostructure leading to the recovering of nonaggregated lipids which can be further analyzed by HPTLC as described in the experimental part. It is worth to note that no peak has been observed on the lane corresponding to the blank solution. Such result allowed us to conclude that peaks corresponding to the analyzed lipids (Egg-PC: Rf = 0.04, PEG_45_-DSPE: Rf = 0.46 and PEG_45_-Tetraether: Rf = 0.79) were not overestimated because of the presence of other peaks having similar Rf values (Figure 3(a)). Calibration curves, based on either peak height or peak area, were plotted for each lipid (Figures 3(b) and 3(c)). From these calibration curves, amounts of lipids contained in each formulation studied were calculated (Table 2) and compared to initial amount of lipids used to prepare liposomes and archaeosomes (Table 2). Results given in Table 2 demonstrated that lipid composition of the prepared liposomes and archaeosomes are very similar to the initial lipid compositions: 88/12 wt% for Egg-PC/PEG_45_-DSPE liposomes instead of an initial composition of 90/10 wt% and 86/14 wt% for Egg-PC/PEG45-Tetraether archaeosomes instead of an initial composition of 90/10 wt%.

### 3.3. Carboxyfluorescein Encapsulation and Release Profile

To assess vesicle stability, the kinetics of encapsulated CF release from PEG-bearing liposomes and archaeosomes was studied at 4°C (standard storage temperature of liposomal formulations) and 37°C (human physiological temperature). The percent release of CF was calculated from the formula described in the experimental part after evaluating the initial amount of encapsulated CF. Thus, a part of the sample containing the vesicle dispersion was treated with triton X-100 [36] for lipid membrane disruption. Then, the fluorescence analysis of the resulting sample allowed us to determine the CF concentration initially entrapped in the nanocarrier using a calibration curve beforehand established.

**Figure 4:**
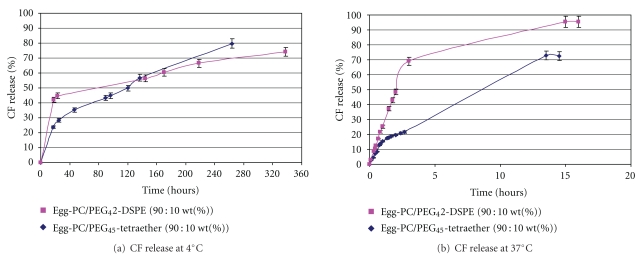
Release (%) of CF from Egg-PC/PEG_45_-Tetraether (90 : 10 wt%) archaeosomes and from Egg-PC/PEG_45_-DSPE (90 : 10 wt%) liposomes at (a) 4°C and (b) 37°C.

The release profile of CF from vesicles at 4°C (Figure 4(a)) showed different rates of leakage between liposome and archaeosome formulations. Indeed, 45% CF release was found to be approximately 20 h for the liposome sample and 100 h for the archaeosome sample. This different behavior was dramatically increased when the formulations were studied at 37°C. As shown in Figure 4(b), there was a rapid leakage of CF from conventional liposomes, where almost 70% of the encapsulated marker was lost within 3 hours. On the contrary, a significant improvement in stability was noted with archaeosomes, which released only 20% during the same period.

Despite their apparent identical characteristics in terms of morphology and surface potential, PEGylated liposomes and archaeosomes exhibited different vesicle stabilities. The presence of only 10 wt% of archaeal tetraether lipid in the liposomal formulations increased significantly the nano-object stability and allowed a slow release of the encapsulated dye at 37°C. This enhanced stability could result from the membrane spanning organization of the PEGylated tetraether lipids within the Egg-PC bilayer membrane, forming a monolayer as previously shown with synthetic cationic tetraethers [13].

## 4. Conclusions

In conclusion, we have demonstrated that small proportions of a novel synthetic PEGylated archaeolipid added to a liposomal formulation increase significantly the nanovector stability and slow down the constant dye release at 37°C. This result is quite promising in so far as a similar behavior could be expected for *in vivo* applications. This study has also shown that HPTLC is a powerful method for analyzing lipid composition. Following such a fundamental work, we have recently evaluated the encapsulation of a therapeutic peptide (anticancer) extracted from marine resources into PEGylated archaeosomes and the *in vivo* efficiency of this peptide-loaded formulation. The first results are very promising and will be published elsewhere. 

## Figures and Tables

**Table 1:** Size (cumulant results), polydispersity (Ip), and zeta potential of prepared formulations. (ND = nondetermined).

Formulation	Size (nm), (Std Dev)	Ip	Zeta potential (mV)
Egg-PC/PEG_45_-DSPE	70 (40)	0.30	−20.0 ± 9
Egg-PC/PEG_45_-Tetraether	80 (30)	0.26	−13.0 ± 6
CF-encapsulated Egg-PC/PEG_45_-DSPE	90 (37)	0.21	Nd
CF-encapsulated Egg-PC/PEG_45_-Tetraether	100 (45)	0.26	Nd

**Table 2:** Amounts of lipids contained in liposomes and archaeosomes calculated from HPTLC data. The given values are an average between peak height and peak area values. The values are reported to a volume of 1 mL.

		Liposome formulations	Archaeosome formulations
Egg-PC	Initial amount (*μ*g)	0.900 (90 wt%)	0.900 (90 wt%)
	*Amount in formulation (*μ*g)*	**0.614 (88 wt%)**	**0.589 (86 wt%) **

PEG_45_-DSPE	Initial amount (*μ*g)	0.100 (10 wt%)	—
	*Amount in formulation (*μ*g)*	**0.087 (12 wt%)**	—

PEG_45_-Tetraether	Initial amount (*μ*g)	—	0.100 (10 wt%)
	*Amount in formulation (*μ*g)*	—	**0.096 (14 wt%)**
